# OMPdb: A Global Hub of Beta-Barrel Outer Membrane Proteins

**DOI:** 10.3389/fbinf.2021.646581

**Published:** 2021-04-09

**Authors:** Ahmed F. Roumia, Konstantinos D. Tsirigos, Margarita C. Theodoropoulou, Ioannis A. Tamposis, Stavros J. Hamodrakas, Pantelis G. Bagos

**Affiliations:** ^1^Department of Computer Science and Biomedical Informatics, University of Thessaly, Lamia, Greece; ^2^EMBL-EBI, Wellcome Genome Campus, Cambridge, United Kingdom; ^3^Section of Cell Biology and Biophysics, Department of Biology, School of Sciences, National and Kapodistrian University of Athens, Athens, Greece

**Keywords:** beta barrel membrane proteins, Gram-negative bacteria, mitochondria, chloroplast, β-barrels, Hidden Markov Model, database, sequence analysis

## Abstract

OMPdb (www.ompdb.org) was introduced as a database for β-barrel outer membrane proteins from Gram-negative bacteria in 2011 and then included 69,354 entries classified into 85 families. The database has been updated continuously using a collection of characteristic profile Hidden Markov Models able to discriminate between the different families of prokaryotic transmembrane β-barrels. The number of families has increased ultimately to a total of 129 families in the current, second major version of OMPdb. New additions have been made in parallel with efforts to update existing families and add novel families. Here, we present the upgrade of OMPdb, which from now on aims to become a global repository for all transmembrane β-barrel proteins, both eukaryotic and bacterial.

## Introduction

Integral membrane proteins (IMPs) play a vital role in cell tasks and communication. IMPs represent roughly 20–30% of the human genome (von Heijne, 1999). They can be structurally divided into two distinct categories, the α-helical membrane proteins and the β-barrel ones (von Heijne, 1999). While the former are commonly found in the bacterial inner membrane and cell membranes of all eukaryotic cells, the latter are located exclusively in the outer membranes of mitochondria, chloroplasts, and Gram-negative bacteria (Cavalier-Smith, 2000). In contrast to the α-helical proteins, which are the major type of the IMPs, the β-barrel membrane proteins are fewer, comprising <3% of the proteins encoded in bacterial genomes (Casadio et al., 2003) (one order of magnitude lower than that of α-helical membrane proteins). However, the β-barrel membrane proteins participate in crucial biological activities in prokaryotic organisms, as well as in the eukaryotic organelles.

Endosymbiosis of Gram-negative bacteria ancestors with host cells generated eukaryotic organelles, chloroplasts (Kleine et al., 2009) and mitochondria (Gray et al., 1999), whose outer membranes contain pores with β-barrel topology. The eukaryotic β-barrel pores are IMPs and can be classified into three main functional groups. Firstly, the specific diffusion channels group, which consists of the voltage-dependent anion channel (VDAC) (De Pinto et al., 2010), the translocase of the outer mitochondrial membrane (TOM40) (Hill et al., 1998), the outer envelope protein 21 (OEP21) (Hemmler et al., 2006), the outer envelope protein 23 (OEP23) (Goetze et al., 2015), the outer envelope protein (OEP37) (Goetze et al., 2006) and the outer envelope protein 40 (OEP40) (Harsman et al., 2016). Secondly, the non-specific diffusion channels group, that includes only the outer envelope protein 24 (OEP24) (Pohlmeyer et al., 1998). Thirdly, the biogenesis/secretion group, that contains the mitochondrial distribution and morphology protein 10 (MDM10) (Meisinger et al., 2004), the sorting and assembly machinery (SAM50) (Stojanovski et al., 2007), the trigalactosyldiacylglycerol 4 (TGD4) (Wang et al., 2012), the translocon at the outer membrane of chloroplasts 75 (TOC75) (Hinnah et al., 2002) and the outer envelope protein 80 (OEP80) (Gross et al., 2020).

**Figure 1:**
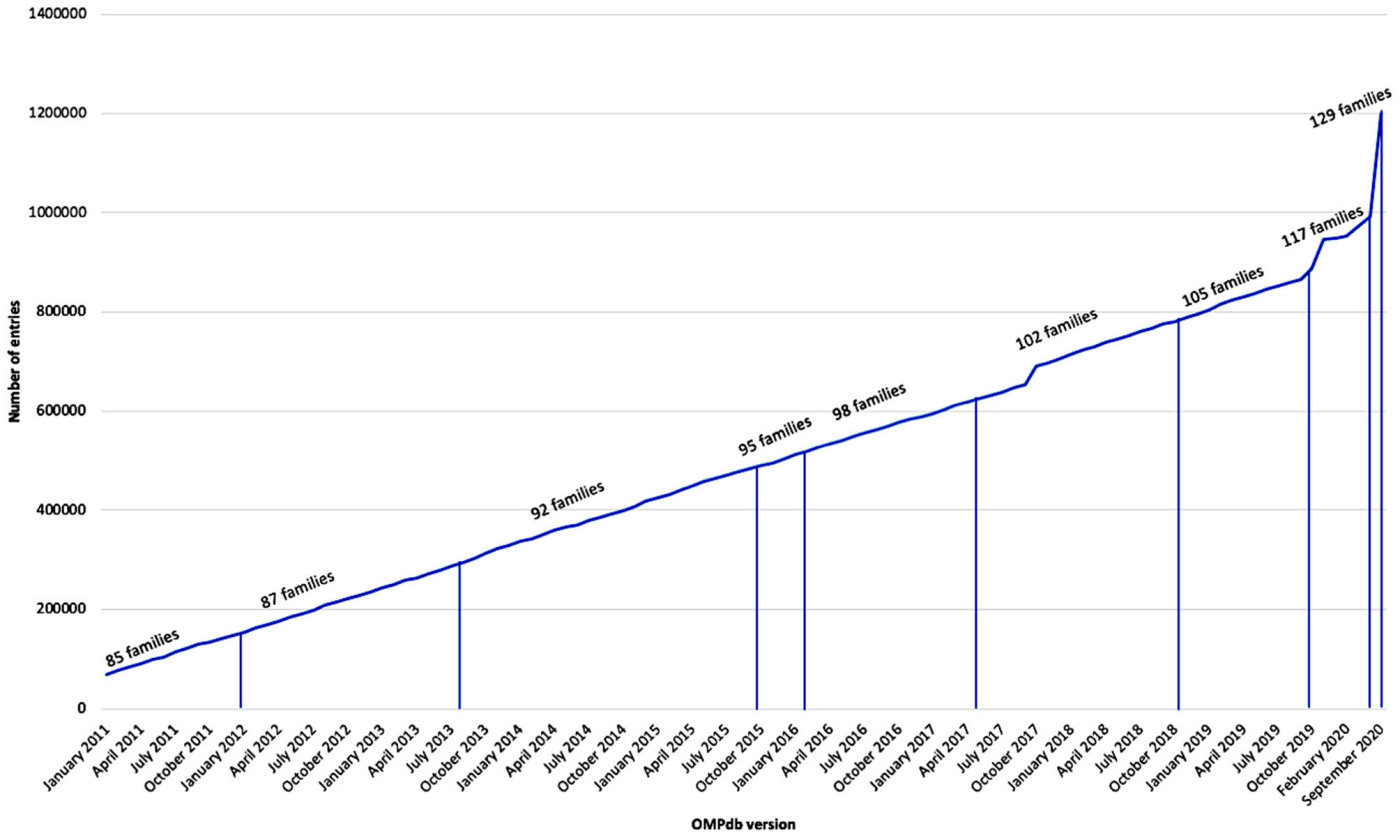
Growth of OMPdb from 2011 till now.

OMPdb is a database of bacterial β-barrel outer membrane proteins (βOMPs). It was launched in 2011 and is until now the most complete collection of Gram-negative bacteria βOMPs. It has been updated continuously using a collection of characteristic profile Hidden Markov Models (pHMMs) as shown in Figure 1.

Up to now, OMPdb (www.ompdb.org) was a repository of only prokaryotic βOMPs and classified them into the following eight functional categories (Tsirigos et al., 2011): receptors, biogenesis/secretion, specific channels, non-specific channels, structural, adhesion, enzymes, and unknown. Specific and non-specific diffusion channels, together with biogenesis/secretion proteins have a crucial role in the bacterial life. Bacterial diffusion channels are the most abundant type of βOMPs in the outer membrane. There are two main classes of bacterial diffusion channels: the non-specific and specific diffusion channels (Schirmer et al., 1995; Koebnik et al., 2000; Schulz, 2002; Nikaido, 2003; Pagès et al., 2008; Wirth et al., 2009; Van Den Berg, 2012). The first class of proteins may have 14 strands, as the outer membrane protein G (OmpG) (Galdiero et al., 2013), 16 strands and permit the flux of hydrophilic substrates smaller than 600 Kda (Ringler and Schulz, 2002) or 26 strands, as the MtrB, the β-barrel member of the outer membrane spanning protein complex, MtrAB (Edwards et al., 2020). The second class members form 12-stranded beta barrels, as in the oligogalacturonate-specific KdgM channel (KdgM) (Wirth et al., 2009), 14-stranded, as the long-chain fatty acid transporter FadL (van den Berg et al., 2004), 16-stranded, like the outer membrane porin B (OprB) (Van Den Berg, 2012) and OprP (Moraes et al., 2007) or 18-stranded, as the outer membrane carboxylate channel (Occ) (Eren et al., 2012; Liu et al., 2012) and maltoporins (Wang et al., 1997; Forst et al., 1998).

In addition to the aforementioned functions of bacterial βOMPs, bacterial adhesion is considered as the first step of biofilm formation and colonization (Dankert et al., 1986). Biofilm, an accumulated biomass of microorganisms, can be harmful to human life where it causes pathogen interaction with host cells (Gristina, 1987; Soto and Hultgren, 1999). Prior to entering inside, pathogenic bacteria adhere to host cells and secrete their products to the host. To this end, bacteria attach to host cells via one of the following three ways: fimbrial (Pili), which is considered the most common proteinous adhesin of Gram-negative bacteria (Soto and Hultgren, 1999) (e.g., *Neisseria gonorrhoeae* Wall and Kaiser, 1999), non-fimbrial adhesions (autotransporters, like e.g., *Haemophilus influenzae* Girard and Mourez, 2006), or using other unique nanofibers like *Caulobacter crescentus* (Corpe, 1972) does. Subsequently, pathogenic bacteria use bacterial secretion as a cellular tool to secrete their virulence factors (predominantly proteins) for invading their host cells. Gram-negative bacteria use eight specific secretion types, with two of them being the most common, i.e., the general secretion (termed the Sec-pathway) and the twin arginine translocation (termed the Tat-pathway Natale et al., 2008; Green and Mecsas, 2016). Despite the high degree of similarity between the targeting signals that direct secretory proteins to their routes, the secretion mechanisms are widely different. The Sec-pathway stimulates the translocation of unfolded proteins, whereupon they fold at the *trans*-side of the membrane into their native structure. On the contrary, the Tat-pathway catalyzes the translocation of folded proteins (Natale et al., 2008).

To reach their proper location, the bacterial βOMPs are being accelerated by the five units (BamA, -B, -C, -D, and -E) of the β-barrel assembly machine (Bam) (Tomasek and Kahne, 2021). BamA, the core of the machine, forms a 16-stranded β-barrel in addition to a periplasmic domain with five polypeptide translocation-associated (POTRA) motifs. It is thought that these five POTRA motifs interact with the four lipoproteins of the Bam complex (Jansen et al., 2015). The Omp85 superfamily of outer membrane proteins encompasses the translocation machines of bacterial βOMPs (BamA) (Voulhoux et al., 2003), mitochondrial βOMPs (SAM50) (Höhr et al., 2018), and chloroplastic ones (TOC75/OEP80) (Day et al., 2019; Gross et al., 2020).

Lipopolysaccharide (LPS) is substantial for the vitality of most Gram-negative bacteria. It plays critical roles not only in the survival of them in severe environments by composing a biofilm but also in colonizing the infected host and avoiding attacks from the human immune system (Zhang et al., 2013; Whitfield and Trent, 2014). LptD, an integral membrane protein, is considered as one of the largest reported bacterial β-barrel so far. Its 26 stranded β-barrel together with LptE form the LPS complex translocon “barrel and plug” at the outer membrane of the Gram-negative bacteria (Dong et al., 2014; Gu et al., 2015).

All Gram-negative bacteria are always in a crucial need for large substrates, such as vitamin B12 or iron-siderophore complexes. Therefore, the outer membrane of Gram-negative bacteria contains diverse high-molecular-weight proteins called TonB-dependent transporter (TBDTs) (Braun, 1995) (e.g., FhuA Coulton et al., 1986) and FusA (Grinter et al., 2016). Furthermore, βOMPs may act as enzymes like Lipopolysaccharides 3-O-deacylase enzyme PagL (Bishop, 2008). Also, βOMPs maintain the stability of the outer membrane as outer membrane protein (OmpA) (Ringler and Schulz, 2002). Lastly, due to the ever-growing number of sequenced genomes of Gram-negative bacteria, the number of proteins with unknown function is relatively small (Tsirigos et al., 2011).

Apart from the typical βOMPs found in the outer membrane of endosymbiotic organelles (e.g., VDAC Bayrhuber et al., 2008, TOC75 Soll and Schleiff, 2004, and OmpG Galdiero et al., 2013), there are also multimeric transmembrane pores formed either from pathogenic microbes in the host's cells, called Pore-forming Toxins (PFTs) (e.g., Aerolysin, which forms heptameric Iacovache et al., 2016 or non-americ Podobnik et al., 2016 pores and Hemolysin De and Olson, 2011), whose role is not yet clear, or from human cells, called Membrane Attack Complex (MAC), that protect human cells by forming pores in the membrane of pathogenic Gram-negative bacteria (Menny et al., 2018).

Because of the biomedical importance of the βOMPs, several biological databases have been launched to annotate and organize the βOMPs in several ways. The Protein Data Bank of Transmembrane Proteins (PDBTM) (http://pdbtm.enzim.hu) was launched in 2004 as the first comprehensive database for experimentally verified transmembrane proteins either α-helical or β-barrels. The database is being updated continuously using the TMDET algorithm. This algorithm can differentiate between the non-transmembrane proteins and transmembrane proteins based on their 3D coordinates. PDBTM (version 2019-02-22) includes 130 non-redundant βOMPs (Kozma et al., 2013).

TMBB-DB (http://beta-barrel.tulane.edu/) was launched in 2012 as a database for transmembrane βOMPs. TMBB-DB includes around 50,000 predicted transmembrane βOMPs (Freeman and Wimley, 2012). To detect the transmembrane βOMPs, the database uses a compilation of two predictors: the Freeman-Wimley algorithm (Freeman and Wimley, 2010) and SignalP5 (Almagro Armenteros et al., 2019).

The Topology Data Bank of Transmembrane Proteins (TOPDB) (http://topdb.enzim.ttk.mta.hu) was launched in 2008 as a hub for the topology of the 3D solved structure transmembrane proteins. TOPDB was dependent on the retrieved data from the Protein Data Bank (PDB) and PDBTM in parallel with the generated topology prediction by HMMTOP (Tusnády et al., 2008). In 2015, they improved the generated topology data by incorporating high throughput techniques like the sequential positions of N- or O-glycosylations. Further, they developed a new algorithm for collecting the scattered topology from various public databases. In addition, they created a new method for evaluating the reliability of the generated topology data. TOPDB provides now the topology information for 201 βOMPs (Dobson et al., 2015).

The Transporter Classification Database (TCDB) (http://www.tcdb.org) is a database of all the transporter proteins. TCDB provides comprehensive structural, functional and medical information for all the transporter proteins. Moreover, TCDB has a specialized software that has been designed and integrated into the TCDB to focus on the distinctive characteristics of the transporter proteins, and subsequently their biomedical function. TCDB classifies 1260 βOMPs into 93 different families (Saier et al., 2016).

The MemProtMD database (http://memprotmd.bioch.ox.ac.uk) was launched in 2015 acting as a repository for the membrane-embedded protein structures and their lipid interactions. MemProtMD provides the molecular dynamics (MD) simulation results of the protein-lipid interactions for 405 βOMPs (Newport et al., 2019).

The Pfam database (http://pfam.xfam.org/) is a widely used repository for protein families. Pfam classifies the protein sequences into clans, families and domains based on specific pHMM for every family. In Pfam, all the βOMP families are gathered together in one clan called MBB (CL0193). This clan contains 92 families with 1,092,329 protein members (El-Gebali et al., 2019).

Here, we present the upgraded and updated OMPdb, a global repository for all βOMPs with experimentally determined 3D structure or not regardless of their location, function and mechanism of pore formation.

## Materials and Methods

**Figure 2:**
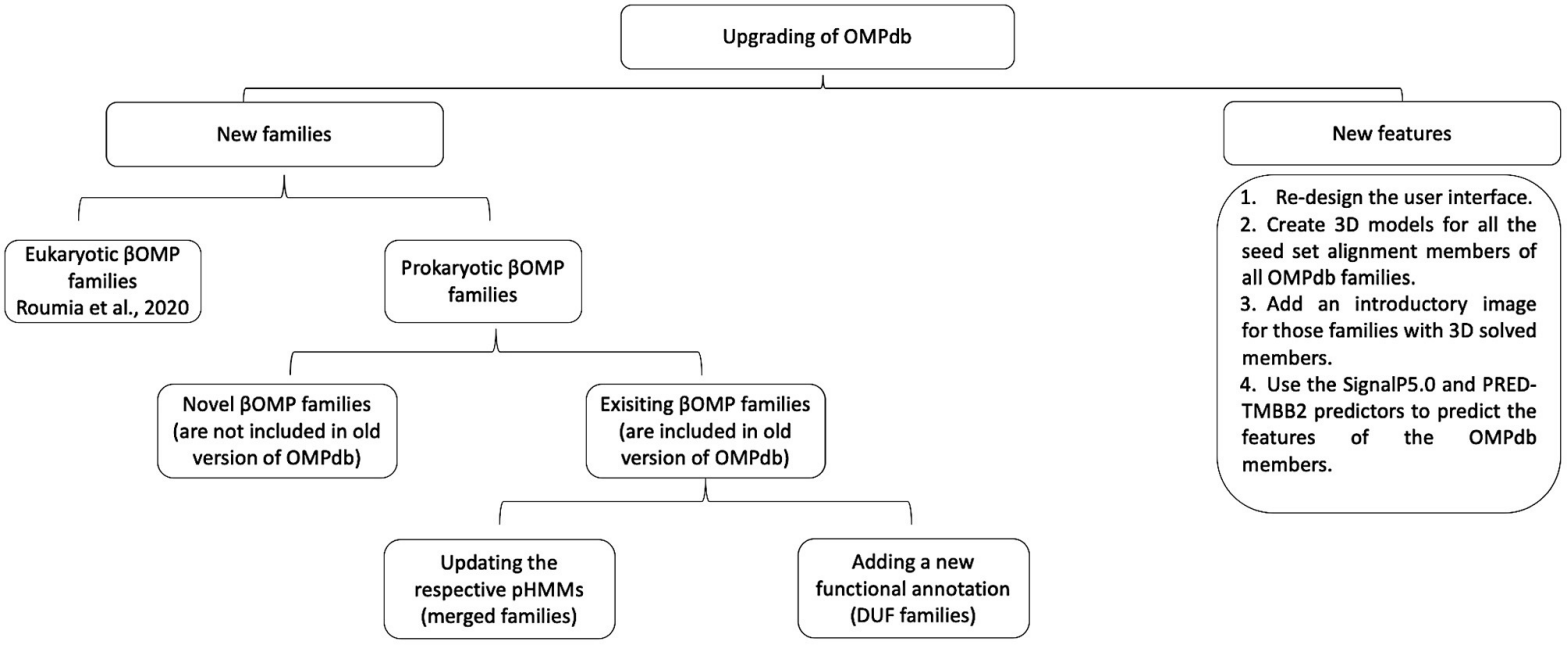
Workflow of the process followed during the upgrade of OMPdb.

In order to upgrade OMPdb, new features and novel βOMPs families, prokaryotic and eukaryotic ones, are included as shown in Figure 2.

### New Families

**Figure 3:**
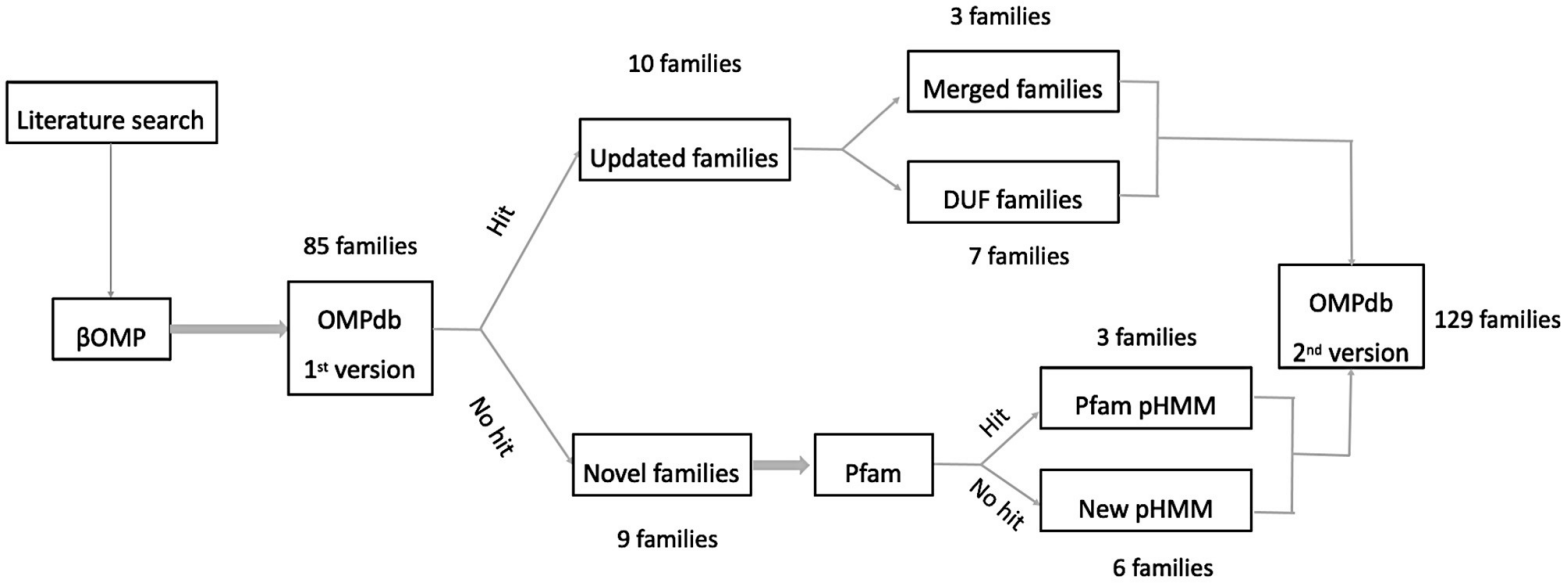
Workflow used for including new prokaryotic βOMPs families into OMPdb.

Initially, our efforts were focused on finding as many new transmembrane βOMPs families in the literature as possible. To this end, we based our literature search along two main axes, adding novel transmembrane βOMPs families and updating current transmembrane βOMPs ones, either by adding new functional annotation or by updating their pHMMs as shown in Figure 3.

First, we performed an extensive literature search to find novel families of transmembrane βOMPs in Gram-negative bacteria (not included in OMPdb Tsirigos et al., 2011). Furthermore, we retrieved all the 3D structures of transmembrane βOMPs that were not included in OMPdb after a thorough search in both PDBTM (version 2019-02-22) (Kozma et al., 2013) and MemProtMD (version 13-10-2019) (Newport et al., 2019). Additionally, we tried to characterize and assign function to as many of the Domain of Unknown Function (DUF) families, members of the MBB clan of Pfam version 33.1 May 2020 (El-Gebali et al., 2019), as possible. It is important to mention here that the same procedure was used in both the first OMPdb publication (Tsirigos et al., 2011) and our recent eukaryotic transmembrane βOMPs publication (Roumia et al., 2020) to create new pHMMs for those families without known domains in Pfam. In brief, the β-barrel domain of the experimentally verified protein(s) reported in the respective published articles were used to perform search against the UniProt (The UniProt Consortium, 2019) database with protein BLAST (Altschul et al., 1997). Subsequently, Multiple Sequence Alignments (MSAs) of the best-scoring results for each family were made by Clustal Omega (Li et al., 2015) and these alignments were refined with MUSCLE (Edgar, 2004). Finally, we built the corresponding pHMMs using the refined alignments with HMMER version 3.3 (Eddy, 1998).

For the Autotransporter and TBDTs families, we updated their pHMMs by adding the new members to their respective Pfam MSAs, and then, following the above-mentioned method. For the Omp85 family we built a new pHMM corresponding only to its bacterial members (BamA/TamA) and mitochondrial ones (SAM50). To this end, we searched Uniprot (The UniProt Consortium, 2019) for mitochondrial SAM50 proteins under Omp85 family, merged the SAM50 search results to the BamA/TamA members, and then, followed the aforementioned steps to building the new pHMM.

Finally, we performed a search with our new pHMMs' collection against the reference proteomes, prokaryotic, and eukaryotic, retrieved from the Uniprot database (Release 2020_03) (The UniProt Consortium, 2019).

### New Features

As part of the upgrade of OMPdb, we decided to include some new features in the latest version. Providing 3D models for all seed set alignment members for all the families included in the database was the first new feature we added. The models were constructed either via homology modeling using SWISS-MODEL (Biasini et al., 2014) (for the families that contained member(s) with experimentally verified structure) or via *de novo* modeling using PHYRE2 (Kelley et al., 2015) (for the families without any determined 3D structures). As an additional feature, we now provide, in each entry, information about the signal peptide and the topology of each βOMP using the SignalP5.0 (Almagro Armenteros et al., 2019) and the PRED-TMBB2 (Tsirigos et al., 2016) tool, respectively.

## Results

### Newly Added Families

In addition to the 11 new eukaryotic transmembrane βOMPs families (Roumia et al., 2020), we included 19 new or updated prokaryotic βOMPs families presented in Table 1. These families are classified into one of the following three categories: novel βOMPs families not included in the old version of OMPdb, known families with new functional annotation or known families with an updated pHMM. Further, for most of the OMPdb families either updated or not, we added 667 recent publications focusing on their respective function.

**Table 1:** Newly added prokaryotic β-barrel families in OMPdb.

**Family^**Category**^**	**Representative member (Uniprot or PDB Acc.)^**References**^**	**Function**	**Pfam domain**
TP0969^1^	R9UUH1 (Cox et al., 2010; Osbak et al., 2016)	Biogenesis/Secretion	No domains found
VP1243^1^	Q87QA7 (Gao et al., 2020)	Active transporter	No domains found
PgaA^1^	4Y25 (Wang et al., 2016)	Biogenesis/Secretion	No domains found
SprA^1^	6H3I (Lauber et al., 2018)	Biogenesis/Secretion	PF14349
Hemolysin^1^	3O44 (De and Olson, 2011)	Non-specific diffusion channel	PF12563; PF07968
Aerolysin^1^	5JZH (Iacovache et al., 2016)	Non-specific diffusion channel	PF01117; PF03440; PF07968
Lysenin^1^	5EC5 (Podobnik et al., 2016)	Non-specific diffusion channel	No domains found
MAC^1^	6H04 (Menny et al., 2018)	Non-specific diffusion channel	PF01823
CsgG^1^	3X2R (Cao et al., 2014)	Biogenesis/Secretion	PF03783
NilB^2^	D3VIY6 (Bhasin et al., 2012)	Adhesion	PF04575
Cj0561c^2^	Q0PAV5 (Guo et al., 2008)	Adhesion	PF11059
COG4313^2^	A5W3Z9 (Belchik et al., 2010; van den Berg et al., 2015)	Specific diffusion channel	PF13557
PorP_SprF^2^	Q11YQ4 (Zhu and McBride, 2014)	Biogenesis/Secretion	PF11751
OMP_b-brl^2^	2LHF (Edrington et al., 2011)	Adhesion	PF13505
TSA^2^	P22940 (Lin et al., 2012; Trung et al., 2019)	Adhesion	PF03249
Oms38^2^	O51379 (Thein et al., 2012)	Non-specific diffusion channels	No domains found
Autotransporter^3^	FabF (5O67) (Rouse et al., 2017) and Hapβ (P45387) (Hendrixson et al., 1997; Meng et al., 2011)	Biogenesis/Secretion	PF03797
TBDTs^3^	4ZGV (Grinter et al., 2016)	Active transporter	PF00593
Omp85^3^	P0A940 (Kim et al., 2007), O32625 (Flack et al., 1995), Q51930 (Ruffolo and Adler, 1996) and Q9K1H0 (Volokhina et al., 2009)	Biogenesis/Secretion	PF01103

1 *This category corresponds to the novel β-barrel protein families which were not included in the previous version of OMPdb*.

2 *This category corresponds to families with a new functional annotation*.

3 *This category corresponds to families for which we built new pHMMs*.

### Novel Families

This first category includes nine novel families presented below.

- TP0969 protein shows structural homology with the TolC protein. TP0969 was predicted as an outer membrane beta barrel by CELLO (Yu et al., 2006), PSORT (Yu et al., 2010), HHomp (Remmert et al., 2009), and PRED-TMBB (Bagos et al., 2004) prediction algorithms and has a signal peptide based on Signal-CF (Chou and Shen, 2007) and SignalP 4.1 (Petersen et al., 2011) prediction algorithms. Furthermore, using mass spectrometry analysis, TP0969 was identified as an outer membrane protein (Cox et al., 2010; Osbak et al., 2016). According to PRED-TMBB2, it forms 12 transmembrane beta strands (Tsirigos et al., 2016).
- *Vibrio parahaemolyticus* is a significant Gram-negative halophilous pathogen. As a result of the consumption of seafood, it may cause a harmful seafood-borne illness in humans. In addition, it causes a wide range of diseases in aquatic animals (Vora et al., 2005; Kawatsu et al., 2006; Datta et al., 2008). VP1243 is a protective antigen which shows a high effective antimicrobial activity against the mentioned organism. VP1243 is widely distributed and highly conserved among the major *Vibrio* Species. VP1243 is considered as a hopeful candidate against the *Vibrio* infections. Western blot analysis revealed that VP1243 is an outer membrane protein (Gao et al., 2020). The first 19 amino acids function as a signal peptide according to SignalP 5.0 (Almagro Armenteros et al., 2019). Further, VP1243 forms 10 transmembrane beta strands based on PRED-TMBB2 prediction (Tsirigos et al., 2016).
- PgaA controls the translocation of de-N-acetylated poly-β-1,6-N-acetyl-d-glucosamine (dPNAG) polymer into the outer membrane of *Escherichia coli* k-12. dPNAG is crucial for biofilm adhesion and has an important role in the maintenance and development of biofilms integration for various bacterial species. The PgaA β-barrel domain (513–807) consists of 16 antiparallel transmembrane β-sheets with β1 and β16 strands interlocking (Wang et al., 2016).
- The Gram-negative *Fibrobacteres-Chlorobi-Bacteroidetes* superphylum has a protein export pathway termed the type 9 secretion system (T9SS), which is considered as an important determinant of pathogenicity in serious periodontal disease (Lasica et al., 2017). These bacteroidetes are famous for the quick and unique gliding motility where their cell surface adhesins move on helical tracks (Shrivastava et al., 2016). Cryo-electron microscopy revealed that SprA is the translocon of T9SS. Furthermore, SprA consists of a large transmembrane β-barrel of 36 strands. The SprA barrel has a lateral entry to the external membrane surface because the barrel pore on the extracellular end is closed (Lauber et al., 2018).
  In addition to the monomeric βOMPs, we also included the multimeric transmembrane βOMPs to the OMPdb families. There are five such families: Hemolysin, Aerolysin, Lysenin, MAC, and CsgG. The first three families act as PFTs (De and Olson, 2011; Iacovache et al., 2016; Podobnik et al., 2016). The MAC family protects the human cells against bacterial attack (Menny et al., 2018) and the CsgG family is involved in protein secretion (Cao et al., 2014).
  PFTs attracted the interest of structural biologists and microbiologists early on due to their ability to act as soluble proteins and transmembrane complexes. PFTs are secreted as water soluble monomers, and later on they connect to the membrane of target cells. There they gather into circular oligomers and enable the insertion into the membrane causing the pore forming and finally the resultant cell death (Anderluh and Lakey, 2008).
- Hemolysin, a heptameric porin, is secreted by *Vibrio cholerae*. The heptamer comprises a ring-like structure with an outer diameter of 135. The channel pore consists of an upper large vestibule formed by β-prism and cytolysin domains, and the stem domains which form a 14-strand β-barrel (De and Olson, 2011).
- Aerolysin is a heptamer where seven monomers form a β-barrel with 14 strands at the outer membrane of *Aeromonas hydrophila*. The aerolysin pore is more stable due its concentric β-barrel fold (Iacovache et al., 2016).
- Lysenin is a PFT of *Eisena fetida* and a member of the aerolysin family. It is a non-amer and forms an 18-strand smooth-walled tubular β-barrel attached to the outside by the C-terminal domains. It is a mushroom-like structure which has a transmembrane pore with a central stem built of a long β-barrel. Having a uniform and stable β-barrel through the entire length of protein makes lysenin an exception among PFTs (Podobnik et al., 2016).
- The MAC is a structure formed at the outer membrane of pathogen cells resulting from activation of the host's complement system. It is one of the immune system's first responders. MAC forms damaging transmembrane channels at the cell membrane of pathogens causing cell lysis and death (Charles A Janeway et al., 2001). It is a multiprotein complex, comprised of six polypeptide chains (C5b, C6, C7, C8α, C8β, and C8γ) together with multiple copies of C9 monomer that are arranged in a split-washer configuration, composing a ring in the membrane which permits free flux of molecules in and out of the cell. The cell dies when enough pores are formed (Sharp et al., 2016). Membrane Attack Complex-Perforin (MACPF) domain (PF01823) is a common domain in all C6-C9 (Tschopp et al., 1986). MAC assembly into the membrane starts when C7 connects to C5b6 (a complex formed by C5b and C6) to compose MAC precursor C5b7 (DiScipio et al., 1988). Subsequently, C8 connects irreversibly to the former complex forming the membrane-inserted C5b8 (Steckel et al., 1983). Then, C5b8 binds to 22 C9 monomers to form C5b9 and polymerizes to finish the MAC pore formation (Podack et al., 1982). In the end, the β-barrel pore is composed after transforming the helical bundles in the MACPF domains into transmembrane β-hairpins through an unknown mechanism (Shepard et al., 1998; Shatursky et al., 1999; Rosado et al., 2007). According to the cryo-EM structure, the formed barrel consists of 88 strands (Dudkina et al., 2016).
- In *Escherichia coli* Xuzhou21, curli subunits are produced and secreted into the outer membrane through the CsgG secretion channel. Curli are a unique group of functional amyloids, which are crucial for host cell adhesion, biofilm formation and colonization of inert surfaces (Barnhart and Chapman, 2006). They are involved in harmful diseases in humans, since they share similar structural and biochemical characteristics with amyloid fibers (Sunde et al., 1997; Moreno-Gonzalez and Soto, 2011). The crystal structure of CsgG showed that it is a symmetric nonameric channel, composed of monomers each having four strands spanning the outer membrane. A 36-stranded β-barrel is formed from nine CsgG monomers. CsgG could perhaps reduce the biofilm formation by controlling Curli secretion and that is why it is studied as a putative antibiotics target (Cao et al., 2014).

### Families With Updated Information

From the literature search, we also were able to assign functional annotation to seven families that had unknown function until now.

- NilB (DUF560) is a surface-exposed outer membrane protein of *Xenorhabdus nematophila*. Expression of NilB is suppressed by NilR and growth in nutrient-rich medium. Members of this family exist in diverse bacteria and are common in the genomes of mucosal pathogens. Bioinformatic analyses reported that NilB is the only characterized member of a family of proteins distinguished by a conserved C-terminal domain of unknown function (DUF560) and N-terminal region tetratricopeptide repeats (TPR). Insertion and deletion mutational analyses revealed that NilB forms a β-barrel with 14 transmembrane strands and seven extracellular surface loops, and an N-terminal globular domain. The globular domain and surface loop 6 play a crucial role in the nematode colonization. Epifluorescence microscopy of these mutants revealed that NilB is necessary at early stages of colonization (Bhasin et al., 2012).
- Cj0561c (DUF2860) is a probable membrane fusion protein and contributes to intestinal colonization. Cj0561c is the only characterized member of DUF2860. Cj0561c is encoded as a membrane transporter gene whose transcription is inhibited by CmeR in *Campylobacter jejuni* (Guo et al., 2008). Based on PRED-TMBB2, Cj0561c is predicted to have 14 beta strands (Tsirigos et al., 2016).
- COG4313 (Phenol_MetA_deg) proteins comprise a huge and widespread family of outer membrane channels and have been involved in the uptake of a set of hydrophobic molecules (van den Berg et al., 2015). The first studied member of COG4313 proteins was the TcpY, an outer membrane protein that eases the uptake of polychlorophenols across the outer membrane of *Cupriavidus necator* JMP134 (Belchik et al., 2010). Pput2725 is another member of the family and forms a 12-stranded barrel at the outer membrane of *Pseudomonas putida* with experimentally verified 3D structure. It is suggested that Pput2725 mediates the uptake of hydrophobic aromatic compounds (van den Berg et al., 2015).
- SprP (PorP_SprF) is associated with the outer membrane of *Cytophaga hutchinsonii*. It is suggested that SprP is involved in protein secretion, a hypothesis made due to its sequence similarity with PorP that is responsible for the secretion of gingipain protease virulence factors in *Porphyromonas gingivalis* type IX secretion system (T9SS) and SprF that is crucial for the delivery of the gliding motility machinery components to the cell surface (Zhu and McBride, 2014). According to PRED-TMBB2, it is predicted to have 14 transmembrane strands (Tsirigos et al., 2016).
- The Outer membrane protein beta-barrel domain (OMP_b-brl) domain is found in a wide range of outer membrane proteins, found mainly in Alphaproteobacteria. The Major Outer Membrane Protein (ompP1) is found in various strains of *Coxiella burnetti* with typical porin properties. It is heat-modifiable and has a channel activity. ompP1 is predicted to form a β-barrel; Omp3b from *Brucella abortus* and homologs from various Alphaproteobacteria that are believed to be involved in bacterial surface control and host cell interactions. In *Pseudomonas aeruginosa*, OprH forms a transmembrane β-barrel protein which consisted of eight strands with four extracellular loops of unequal size. Moreover, *in vivo* and *in vitro* biochemical studies revealed that OprH interacts with LPS in *P. aeruginosa* outer membranes (Varghees et al., 2002; Edrington et al., 2011).
- The Type Specific Antigen (TSA) consists of several antigenic variants in the genera *Rickettsia* and *Orientia* that belong to the family *Rickettsiaceae*. *Rickettsiaceae*, intracellular bacteria, are the causative agent of the Rickettsioses which are an arthropod-borne zoonoses. The TSA of 56-kilodaltons located on the rickettsial surface is responsible for the variation. *Orientia tsutsugamushi* are the causative agent of scrub typhus, is an obligate intracellular pathogen. In *O. tsutsugamushi*, there are three major outer membrane proteins: TSA56, TSA47, and TSA22. The TSA56 is the major outer membrane protein responsible for *O. tsutsugamushi* adhesion (Lin et al., 2012; Trung et al., 2019).
- Relapsing fever is a worldwide, endemic disease caused by several spirochetal species belonging to the genus *Borrelia*. Oms38 (outer membrane-spanning protein of 38 kDa) is present in the outer membranes of *B. duttonii, B. hermsii, B. recurrentis*, and *B. turicatae* as well. Characterization of Oms38 was performed using the black lipid bilayer method, which demonstrated that Oms38 forms small, water-filled channels of 80 pS in 1 M KCl that did not exhibit voltage-dependent closure. The Oms38 channel is slightly selective for anions and shows a ratio of permeability for cations over anions of 0.41 in KCl. Analysis of the deduced amino acid sequences revealed that Oms38 contains an N-terminal signal sequence which is processed under *in vivo* conditions. Oms38 is highly conserved within the four studied relapsing fever species, sharing an overall amino acid identity of 58% and with a strong indication for the presence of a ß-barrel formation (Thein et al., 2008). DipA (BB_0418) forms a specific porin for dicarboxylates. It is suggested that DipA plays a crucial role for the flux of specific nutrients toward different *Borrelia* species (Thein et al., 2012).

### Families With Updated pHMMs

During the update of OMPdb, we identified proteins with the same biological function as two pre-existing beta barrel families, namely the Autotransporter (PF03797) and the TBDTs (PF00593) families. Interestingly, the Pfam profiles were unable to detect these proteins, so we updated the respective profiles.

For the Autotransporter family, two new members were added in the seed set, the functional amyloid transporter from *Pseudomonas*, FabF (PDB: 5O67) (Rouse et al., 2017), and the *Haemophilus influenzae* Hap β-barrel domain, Hap_β_ (Uniprot: P45387) (Hendrixson et al., 1997; Meng et al., 2011), and the pHMM was rebuilt. FapF is a new class of secretion system similar to type V secretion in the autotransporter proteins (AT-1). FapF is characterized by a C-terminal, 12 β-stranded, helix-blocked pore in the closed state. In contrast to other autotransporters which completely pass the pore, the N terminus of the truncated FapF structure exits the barrel on the periplasmic side (Rouse et al., 2017). Hap_β_ is the β-barrel domain of self-associating autotransporters (SAATs). SAATs is a set of virulence factors that enhance bacterial biofilm formation (Klemm et al., 2006). The *Haemophilus influenzae* Hap SAAT consists of three domains: residues 1–25 correspond to the signal peptide, residues 26-−1036 refer to the Hap_s_ called the passenger domain, and residues 1037−1394 form the 14 β-stranded barrel at the C-terminal (Hendrixson et al., 1997; Meng et al., 2011).

TBDTs pHMM was updated by adding the respective members of FusA in the seed set. FusA is used by the phytopathogenic *Pectobacterium* spp. as a new class of TBDTs. FusA is a 22-stranded transmembrane β-barrel responsible for importing pectocin and ferrodoxin in *Pectobacterium* spp. (Grinter et al., 2016).

Finally, we created a new pHMM for BamA/TamA and SAM50 families. Previously, BamA/TamA and SAM50, along with TOC75 and OEP80 constituted the Omp85 family (PF01103). Since we were able to create two separated chloroplastic pHMMs for TOC75 and OEP80 (Roumia et al., 2020), we decided to build the new pHHM and now we have the ability to easily distinguish members of the three eukaryotic families (TOC75, OEP80, and SAM50) when searching against eukaryotic proteomes.

**Figure 4:**
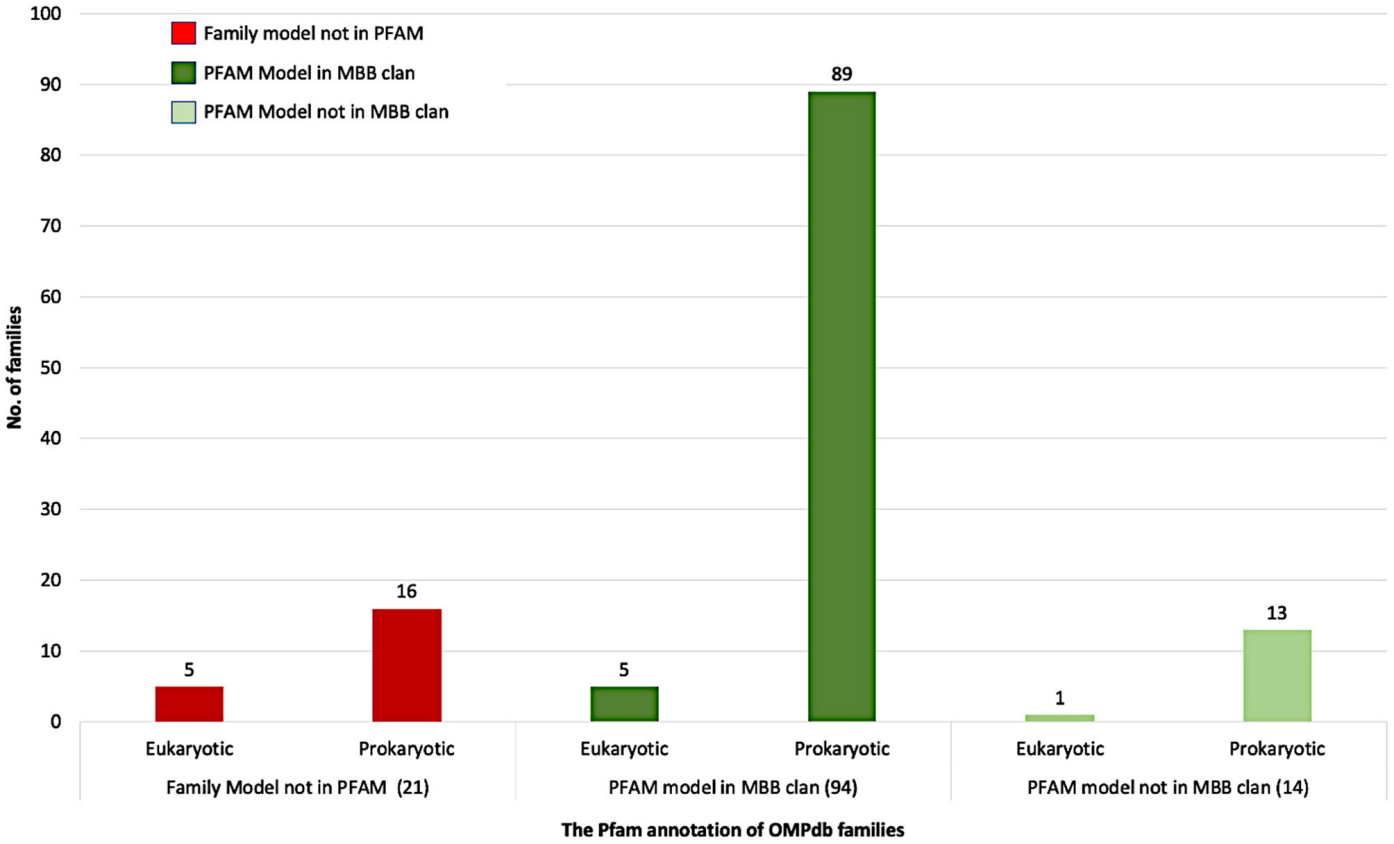
The Pfam annotation of OMPdb β-barrel protein families.

Figure 4 summarizes the Pfam annotation of the 129 families in OMPdb. 72.9% of the families belong to the outer membrane beta-barrel protein superfamily (MBB clan), while 10.8% of the families are in Pfam but not in MBB clan. Interestingly enough, for 16.3% of OMPdb families, there was no information at all in the Pfam database.

### Search Using the OMPdb pHMMs' Collection Against Reference Proteomes

**Figure 5:**
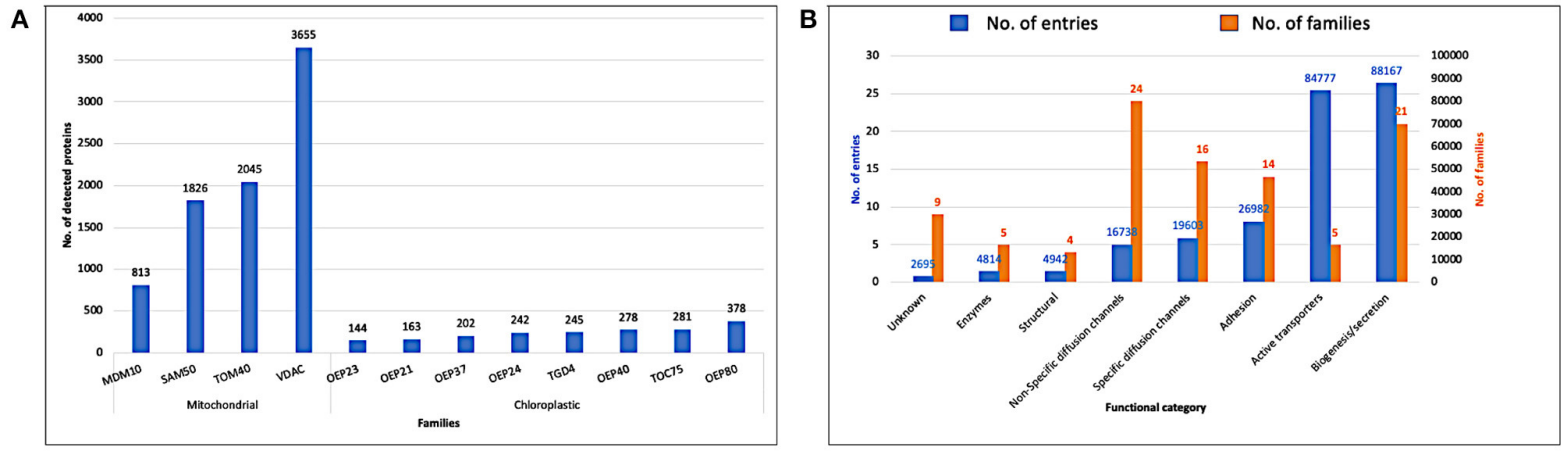
**(A)** Detected proteins by the eukaryotic pHMM library against eukaryotic reference proteomes (Uniprot release 2020_03). **(B)** Detected proteins by prokaryotic pHMM library against bacterial reference proteomes (Uniprot release 2020_03).

In Figure 5, we display the detected proteins by the two pHMM libraries, eukaryotic and prokaryotic, against the retrieved reference proteomes from Uniprot (Release 2020_03). The eukaryotic pHMM library detected at least one protein in 96.97% of the eukaryotic reference proteomes and the number of detected proteins for each family is depicted in Figure 5A. As shown in Figure 5B, the detected prokaryotic proteins were distributed functionally into eight categories. Most of them belong to the receptor category followed by the biogenesis/secretion category, while the enzymes category shows the lowest number of detected proteins.

**Figure 6:**
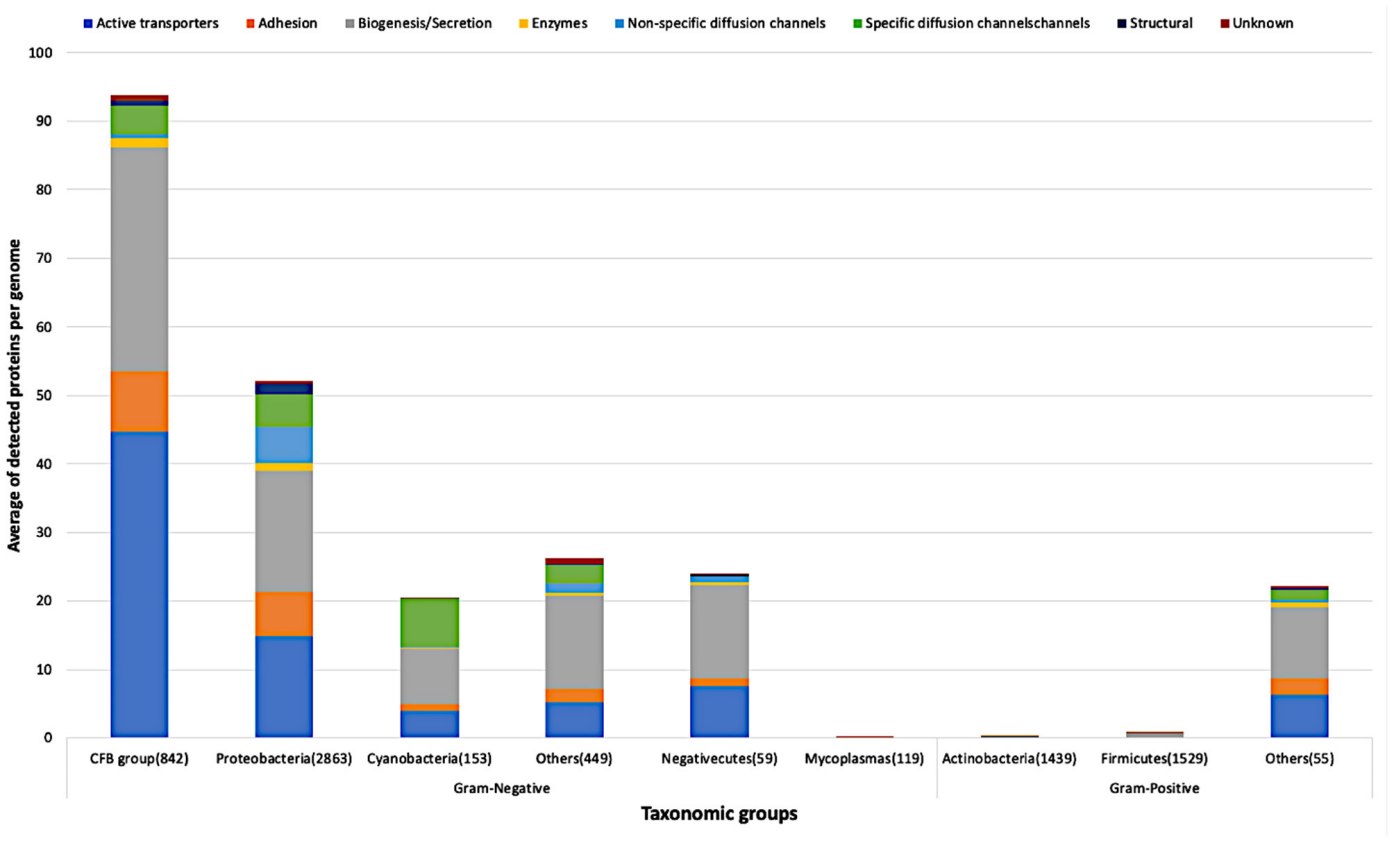
Average of detected functional proteins per genome for the different bacterial taxonomic groups.

As a next step, we followed the taxonomy to classify the bacterial reference proteomes according to the NCBI Common Taxonomy Tree (Sayers et al., 2009). The bacterial reference proteomes are classified into 16 main taxonomic groups, three groups from Gram-positive bacteria, *Actinobacteria, Firmicutes* and *other bacteria*, and the rest 13 groups from Gram-negative bacteria. As shown in Figure 6, Gram-negative bacteria are classified into six main groups: *Cytophaga, Fusobacterium*, and *Bacteroides group* (CFB group), *Proteobacteria, Cyanobacteria, Mycoplasmas, Negativecutes*, and *other bacteria*. All taxonomic groups that account for <1% of the total reference proteomes in both classes, Gram-positive or Gram-negative bacteria, are classified under “other bacteria.”

*CFB* group has the highest average of detected proteins followed by the *Proteobacteria* group of Gram-negative bacteria. In line with what we expected, *Actinobacteria* and *Firmicutes*, the two main Gram-positive bacteria taxonomic groups, have few representatives of the above-mentioned functional categories. Nevertheless, it is clear that the *Firmicutes* group has representatives relatively more than the *Actinobacteria* group as shown in Figure 6.

**Figure 7:**
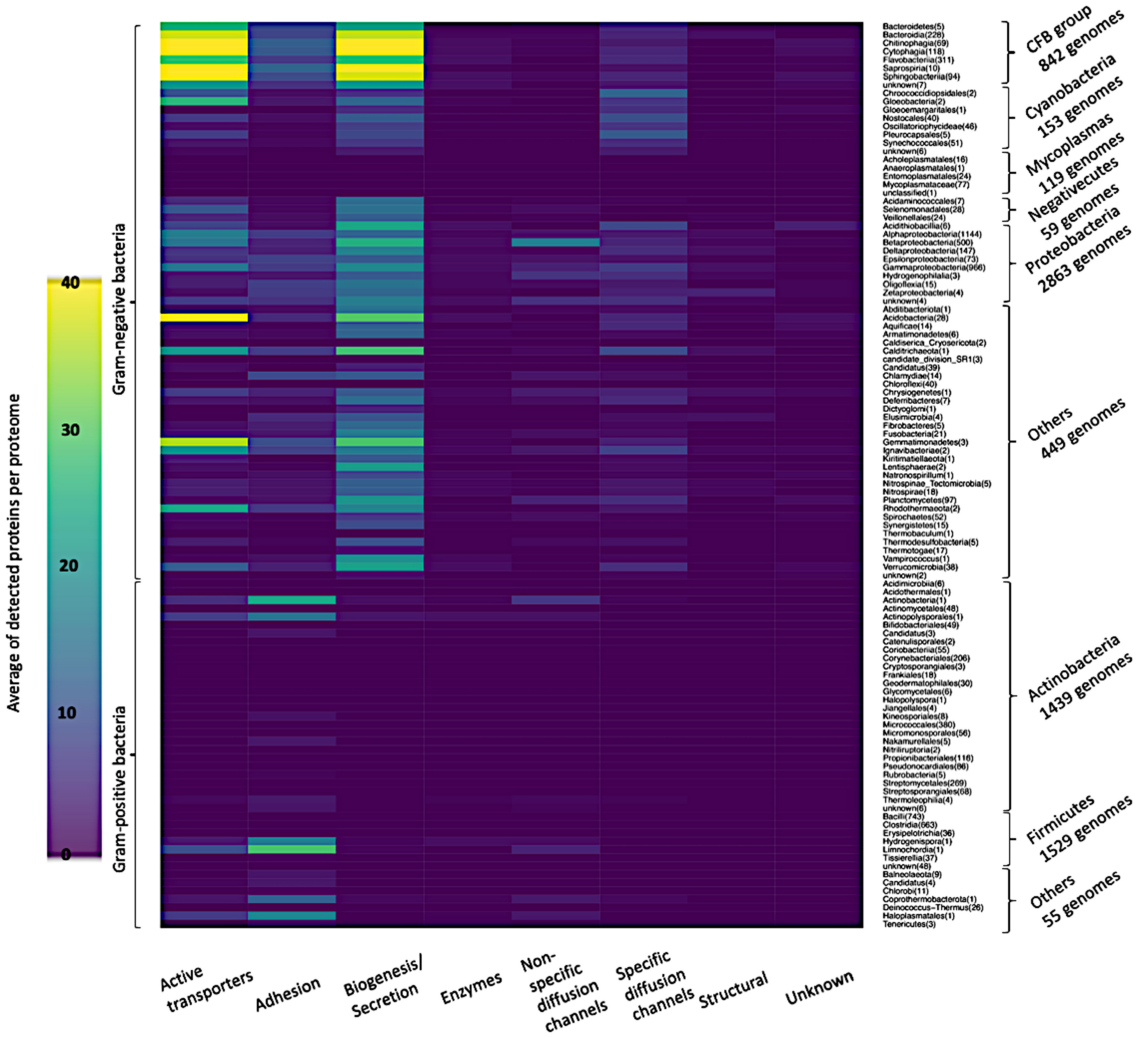
Heatmap of the average of detected functional proteins in all bacterial taxonomic groups.

Figure 7 shows in detail the average of detected functional proteins for all bacterial taxonomic groups either Gram-negative or Gram-positive ones. Except for the *Mycoplasmas* group, all Gram-negative bacteria taxonomic groups have a significant number of representatives of the active transporters, biogenesis/secretion, specific diffusion channels, and unknown function categories. For the *Cyanobacteria* group, the non-specific diffusion channels category is almost absent as shown in Figure 7.

### New Features

Beside re-designing the user interface, we now provide structural information through 3D models for all seed set members of each family and this feature gives deep and concise information about the respective family. In total, we provide 3,264 models for families with at least one member with experimentally verified crystal structure and 2,116 models for families without any previous knowledge about their 3D structure. In addition, we provide an introductory structure image structure at the main page of the respective family, for every family including members with experimentally verified 3D. Moreover, for all the OMPdb entries, we present the predicted features of the signal peptide and β-barrel topology using SignalP5 and PRED-TMBB2 predictors.

## Discussion

In this work, we presented the new content and updated features of OMPdb, a global hub for all βOMPs using 128 characteristic pHMMs. This collection of pHMMs includes 11 eukaryotic and 117 prokaryotic families in addition to the *Treponema pectinovorum* ATCC 33768 mompA family (Walker et al., 1997) that lacks a pHMM due to missing known homologues of its representative member.

OMPdb in its second version, still has a clear advantage against all the other databases that contain βOMPs. As shown in Table 2, OMPdb is currently the most complete resource for transmembrane β-barrels, since it has the highest number of protein and family entries, provides numerous literature references, offers sequence annotation, cross-references with many related databases and the ability to use search and prediction tools. Due to the discontinuation of TMBETA GENOME (Gromiha et al., 2007), PRNDS (Katta et al., 2004), HHompDB (Remmert et al., 2009), and TMPDB (Ikeda et al., 2003) databases since the first version of OMPdb, we excluded them from the comparison. In contrast, we included in the comparison two new databases, Mpstruc (White, 2009) and MemprotMD (Newport et al., 2019).

**Table 2:** A comparison between OMPdb and other databases that include β-barrel proteins.

**Database Features**	**OMPdb (current work)**	**Pfam V. 33.1 (MBB clan) (El-Gebali et al., 2019)**	**TCDB (Saier et al., 2016)**	**PDBTM (Kozma et al., 2013)**	**Mpstruc (White, 2009)**	**MemprotMD (Newport et al., 2019)**	**TOPDB (Dobson et al., 2015)**	**ePSORTdb (Peabody et al., 2016)**	**OPM (Lomize et al., 2012)**	**MPdb (Raman et al., 2006)**
Total entries	1198558	1092329	1260	130	283	405	201	839	291	153
Total families	129	92	93	-	17	-	-	-	34	29
Data retrieval	Semi-automated	Semi-automated	Manual	Semi-automated	Automated	Automated	Semi-automated	Manual	Semi-automated	Manual
Literature references	+	+	+	–	+	+	+	+	–	+
Signal peptide/TM segments annotation	+/+	–/–	–/–	±	–	–	+/+	–/–	±	–/–
Number of databases cross-referenced	13	5	14	2	3	6	3	2	4	2
Prediction/search tools	BLAST/HMMER/PRED-TMBB2/Text search	Text search/HMMER	MEMSAT/HHrepID/AveHAS/HMMTop/Text search	Text search	Text search/Fuzzy search	Text search	Text search	Psortb/BLAST	Text search	Text search

Based on whether a family exists in Pfam, and specifically if it is a member of the MBB clan, the OMPdb families can be classified into three categories as shown in Figure 4. The first one includes 94 families belonging to the MBB clan in Pfam. In this category, we updated the pHMMs of MDM10 (PF12519), TBDTs (PF00593) and Autotransporter (PF03797), while the Porin_3 (PF01459) pHMM was replaced with two new pHMMs, VDAC, and TOM40 (Roumia et al., 2020). Furthermore, the pHMM of Omp85 (PF01103) was updated to contain only BamA/TamA and SAM50 proteins due to having two specific pHMMs for TOC75 and OEP80 families (Roumia et al., 2020). The second category refers to 14 families which are in Pfam but not in the MBB clan. Although Hemolysin and SprA families had Pfam pHMMs, we updated their respective pHMMs. For Hemolysin, the respective pHMM (PF12563) did not cover all the transmembrane barrel region, while, in the SprA family (PF14349), the transmembrane barrel part was intermittent. Therefore, we built two novel pHMMs for Hemolysin and SprA families. The third category (families not included in Pfam), consists of 5 eukaryotic (Roumia et al., 2020) and 16 prokaryotic families. Interestingly, for these 21 families, there is no information in Pfam whatsoever and these novel families can only be found in OMPdb.

In OMPdb we focus on offering as much information about the nature of the transmembrane barrel part in each family to the user as possible. To this end, we re-designed the OMPdb user interface to include various new features. The first one is adding an introductory structure image for 44% of the OMPdb families that presents to the user the β-barrel features of these families at one glance.

The 3D structure dictates the biological function of every protein. Knowing the 3D structure of a protein provides a higher level of understanding of its molecular action. This knowledge allows us to create hypotheses on controlling, modifying or affecting on this protein. We now provide a 3D model for each of the seed set members of every OMPdb family. For the families including members with experimentally verified 3D structure (57 families), we constructed the models using homology modeling via the SWISS-MODEL server (Biasini et al., 2014). We provide the used template as the representative structure for each family. We used *de novo* modeling through PHYRE2 (Kelley et al., 2015) for the rest of the families (71 families).

The nascent βOMPs either eukaryotic (Rapoport, 2007) or prokaryotic (Nouwen et al., 2007) have signal peptides to be recognized by the Sec or Tat pathways. After or during the assembly of the transmembrane proteins, the signal peptides are being cleaved by signal peptidase (SPase). The Tat substrates can be cleaved by SPase I or II while the Sec substrates can be processed by SPase I, II, or III. SignalP5 is the first predictor that provides a deep neural network-based method for predicting the signal peptide cleaved by (Sec/SPI), (Sec/SPII), and (Tat/SPI) (Almagro Armenteros et al., 2019). PRED-TMBB2 is a predictor for the β-barrel topology based on Hidden Markov Models. It excels the best available β-barrel predictor by 7% (Tsirigos et al., 2016) (Tsirigos et al., 2016). Both predictors are therefore used to provide the topological features of each protein.

For the detection of βOMPs in the reference proteomes, we used the collection of OMPdb's pHMMs after excluding all the DUF families (19 families) that do not have any literature information regarding their function (Supplementary Table 1). It is worth noting that the updated Omp85 pHMM was used twice, in both the eukaryotic library to detect the SAM50 members and the prokaryotic library to detect the BamA members.

Very few proteins were detected in the *Mycoplasmas* group, although they are Gram-negative bacteria, and the reason is the absence of a cell wall in this taxonomic group (Cimolai, 2001) (Figures 6, 7). On the other hand, the *Firmicutes* group, a group of Gram-positive bacteria, shows a significant number of detected proteins. *Firmicutes* is a bacterial phylum, in which most of its organisms are Gram-positive bacteria. However, *Firmicutes* contains a class called *Negativecutes*, which is classified as Gram-negative bacteria (Sutcliffe, 2010). Interestingly enough, around 51.67% of the detected proteins at the *Firmicutes* group (1588 proteomes) belong to the 59 *Negativecutes* proteomes.

Our analysis showed that the biogenesis/secretion category was the largest one in comparison with the other functional categories The Outer membrane Factor family (OMF) comprises 40.5% of all detected proteins. These proteins are involved in the type I secretion pathway (Sec-independent), which is widespread and permits the secretion of proteins of different sizes and functions using uncleaved secretion signal at the C-terminal (Delepelaire, 2004). Around 9% of all detected proteins belong to each of the following families: Secretin, Autotransporter (AT), *Porphyromonas gingivalis* PorT and Outer Membrane Protein Insertion Porin (OmpIP/Omp85). The members of these four families are involved in type II and type III secretion pathway (Collins et al., 2004), type V secretory pathway (Desvaux et al., 2004), secretion of gingipains from *Porphyromonas gingivalis* (Nguyen et al., 2009) and the translocation of βOMPs into the outer membrane of Gram-negative bacteria (Noinaj et al., 2015), respectively. As for the active transporters category, it consists of five families (Supplementary Figure 2). It should be noted that the TBDTs family detects around 99.83% of all active transporter proteins. The aforementioned family represents the TBDTs transport system, which is involved in the active transport mechanism of nutrients through the outer membrane of Gram-negative bacteria (Higgs et al., 2002). It is noteworthy that the existence of active transporters, biogenesis/secretion, specific diffusion channels, and non-specific diffusion channels categories in all taxonomic groups of Gram-negative bacteria are in line with the findings of Nikaido (Nikaido, 2003), who revealed that the general part of βOMPs is acting as channels and transporters. The only inconsistency is the absence of non-specific diffusion channels in the *Cyanobacteria* group. The adhesion to the host cell is the first step bacteria take to establish a successful infection. The bacterial adhesion may occur directly via monomeric adhesins (Soto and Hultgren, 1999) or via highly advanced machines like the type III secretion systems (Autotransporter) (Pizarro-Cerdá and Cossart, 2006), which belongs to the biogenesis/secretion category, or by using special nanofibers (Corpe, 1972). Although there are multiple ways of bacterial adhesion (e.g., autotransporter and special nanofibers), there are 14 families for which their main function is adhesion. The OMP_b-brl forms around 72.74% of the detected adhesion proteins. The representative member of the aforementioned family, OprH, is involved in the stability of the outer membranes of Gram-negative bacteria by interacting with the LPS (Edrington et al., 2011) (Figure 7 and Supplementary Figure 3).

The widespread existence of specific and non-specific diffusion channels in all Gram-negative bacteria reflects the effective role of porins on the bacterial cell life. In addition to acting as passages for the nutrients into the Gram-negative bacteria, porins are affecting the bacterial pathogenicity due to their high prevalence at the bacterial surface structure (Naumann et al., 1999; Achouak et al., 2006; Galdiero et al., 2013; Choi and Lee, 2019) (Supplementary Figures 4, 5). In *Cyanobacteria*, the absence of the non-specific diffusion channels category reflects the low permeability of the cyanobacterial outer membrane (Figures 6, 7). The reason why the cyanobacterial outer membranes are highly impermeable is to prevent the flux of toxic compounds into the cell and this is an advantage for the cyanobacterial autotrophic lifestyle (Kowata et al., 2017). In the structural category (Supplementary Figure 6), the OmpA family counts around 71.35% of the proteins. This high percentage gives consideration to the critical effect of this domain on the structural strength and shape of bacterial cells (Höltje, 1998), due to the non-covalent interaction between the respective domain with the peptidoglycan layer (Grizot and Buchanan, 2004).

As expected, the enzyme category has the lowest number of detected proteins, because only a limited number of βOMPs act as enzymes (Bishop, 2008). 44.69% of the enzyme proteins belong in the Outer membrane-localized lipid A 3-O-deacylase (PagL) family that is widespread among Gram-negative bacteria (Rutten et al., 2006) (Figure 7, Supplementary Figure 7).

Due to the significant progress that has been made toward the annotation of Gram-negative bacteria genomes, the “unknown” category comprises only around 1.08% of our analysis (Figure 7, Supplementary Figure 8). Around 22% of Pfam proteins belong to DUF families (El-Gebali et al., 2019). Generally, there is an enormous number of proteins with unknown function and the reason is the difficulty of assigning function to proteins, which is a major goal of structural biology, biochemistry and bioinformatics (McKay et al., 2015).

## Conclusions

The OMPdb database is a thorough, up-to date and continuously updated βOMPs database. OMPdb, in its second version, contains two different libraries of pHMMs that cover all the kinds of βOMPs. As of September 2020, it has around 1.2 million entries whose domains are classified into 129 different β-barrel families either prokaryotic or eukaryotic ones. OMPdb operates for more than 10 years and will be continuously updated in the future. The database participates in ELIXIR-GR, the Greek National Node of the ESFRI European RI ELIXIR, a distributed infrastructure that will allow the life science research community across Europe to share and store their research data as part of an organized network, whereas, at the same time is in close collaboration with other specialized protein databases (Babbitt et al., 2015; Holliday et al., 2015).

## Data Availability Statement

The original contributions presented in the study are included in the article/Supplementary Material, further inquiries can be directed to the corresponding author/s.

## Author Contributions

PB conceived the presented idea. PB, KT, and MT conceived and planned the experiments. AR carried out the experiments and wrote the manuscript with support from MT and KT. All authors provided critical feedback and helped shape the research, analysis, and manuscript.

## Conflict of Interest

The authors declare that the research was conducted in the absence of any commercial or financial relationships that could be construed as a potential conflict of interest.

## Abbreviations

βOMPs: β-barrel outer membrane proteins.
