# Supplementary material for: OMPdb: A Global Hub of Beta-Barrel Outer Membrane Proteins
Source: Front Bioinform. 2021 Apr 9;1:646581. doi: 10.3389/fbinf.2021.646581 (PMC9581022; doi:10.3389/fbinf.2021.646581)
Supplement: Supplementary file 1 [file Data_Sheet_1.PDF]

Supplementary Material

1 Supplementary Figures and Tables

1.1 Supplementary Figures

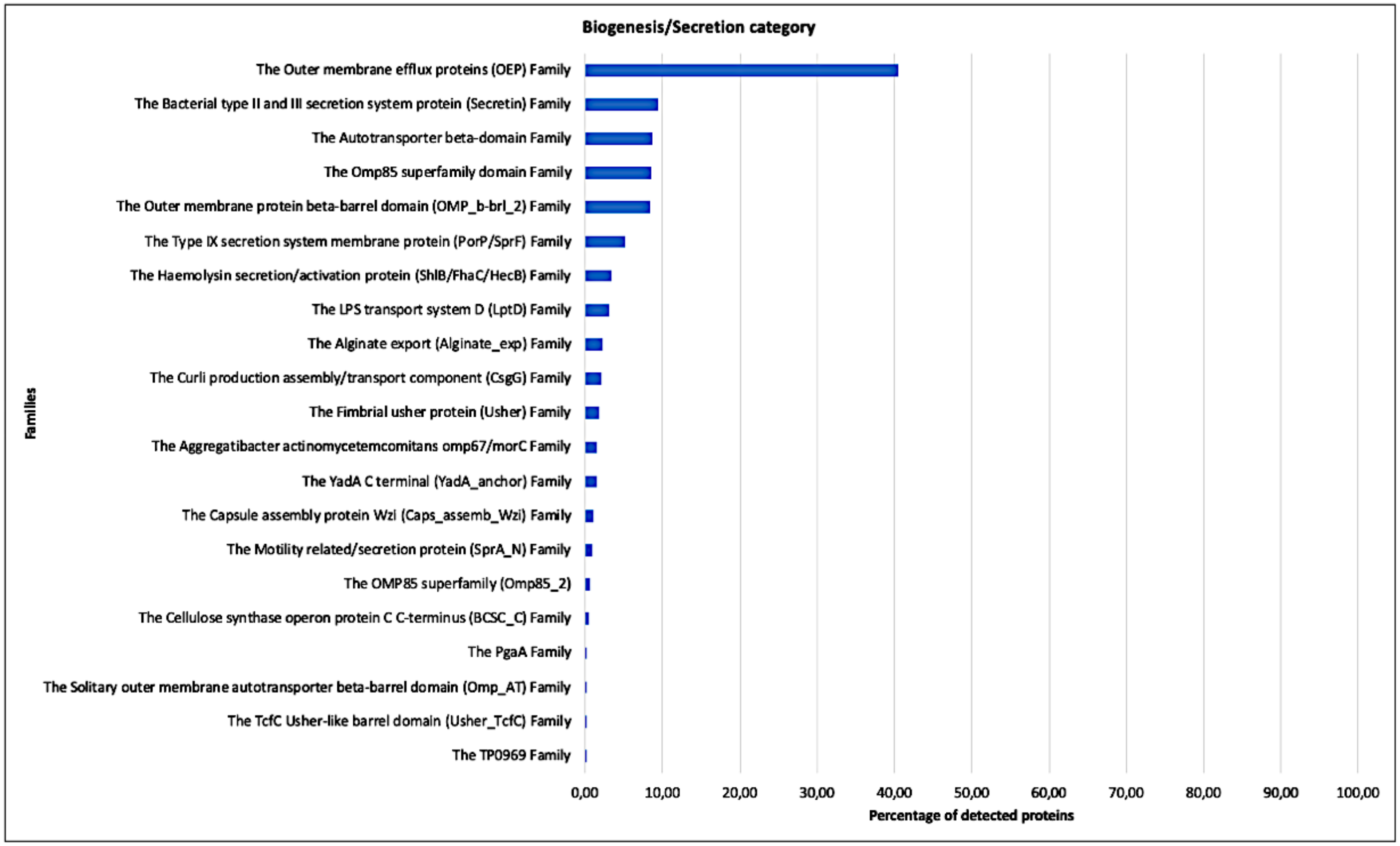

Supplementary Figure 1. The fraction of detected proteins in each of the families belonging to ‘biogenesis/secretion’ category.

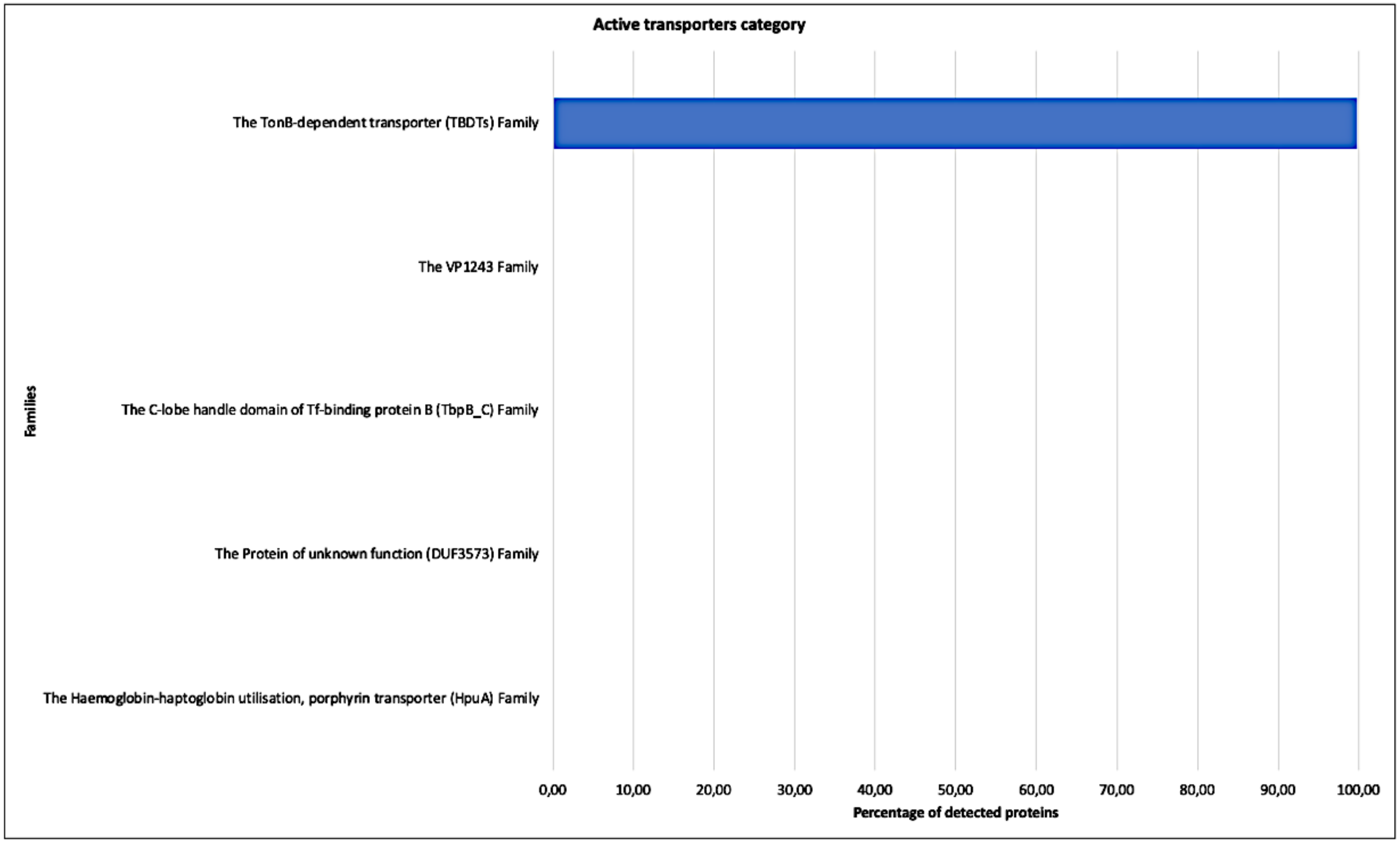

Supplementary Figure 2. The fraction of detected proteins in each of the families belonging to ‘active transporters’ category.

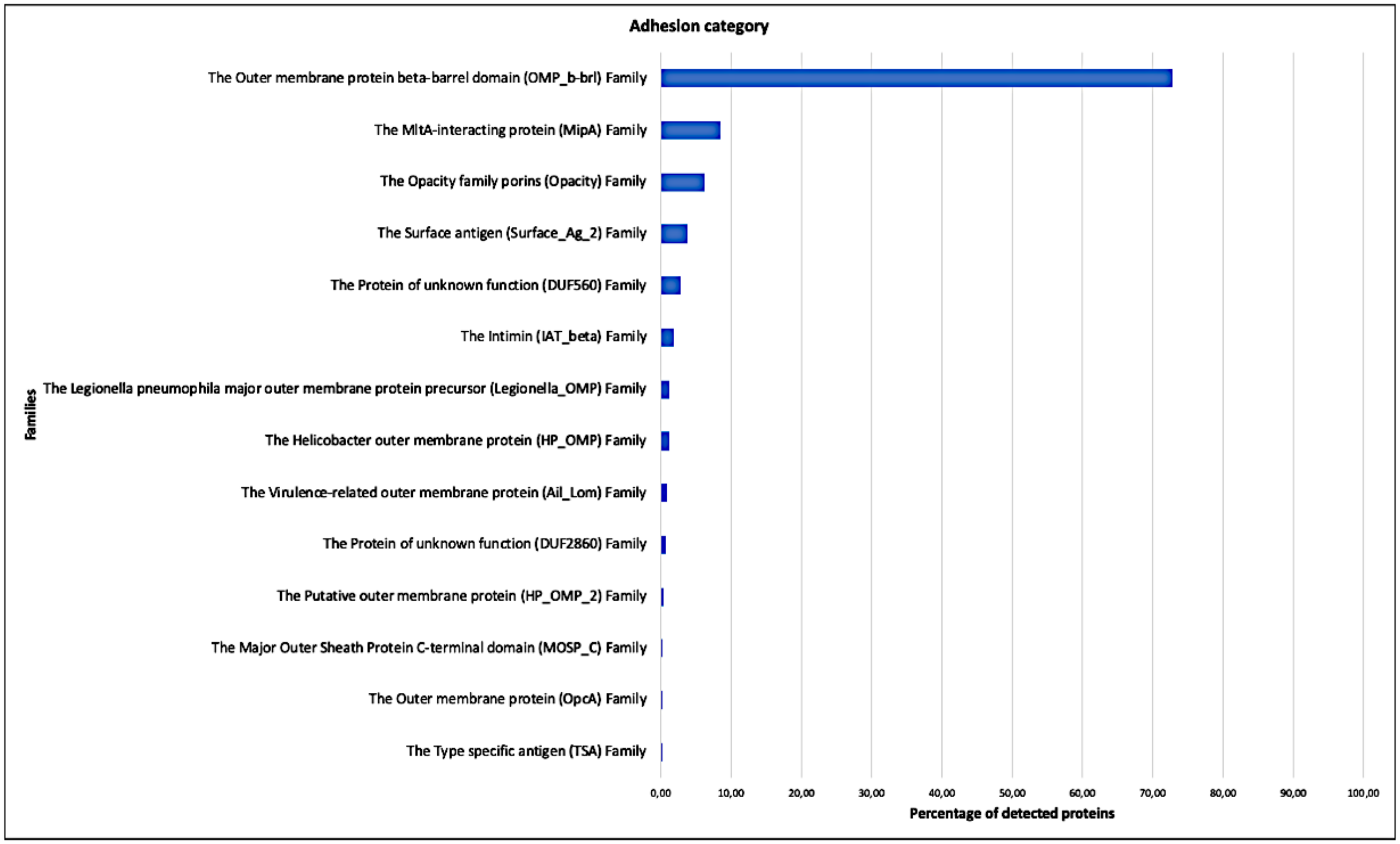

Supplementary Figure 3. The fraction of detected proteins in each of the families belonging to ‘adhesion’ category.

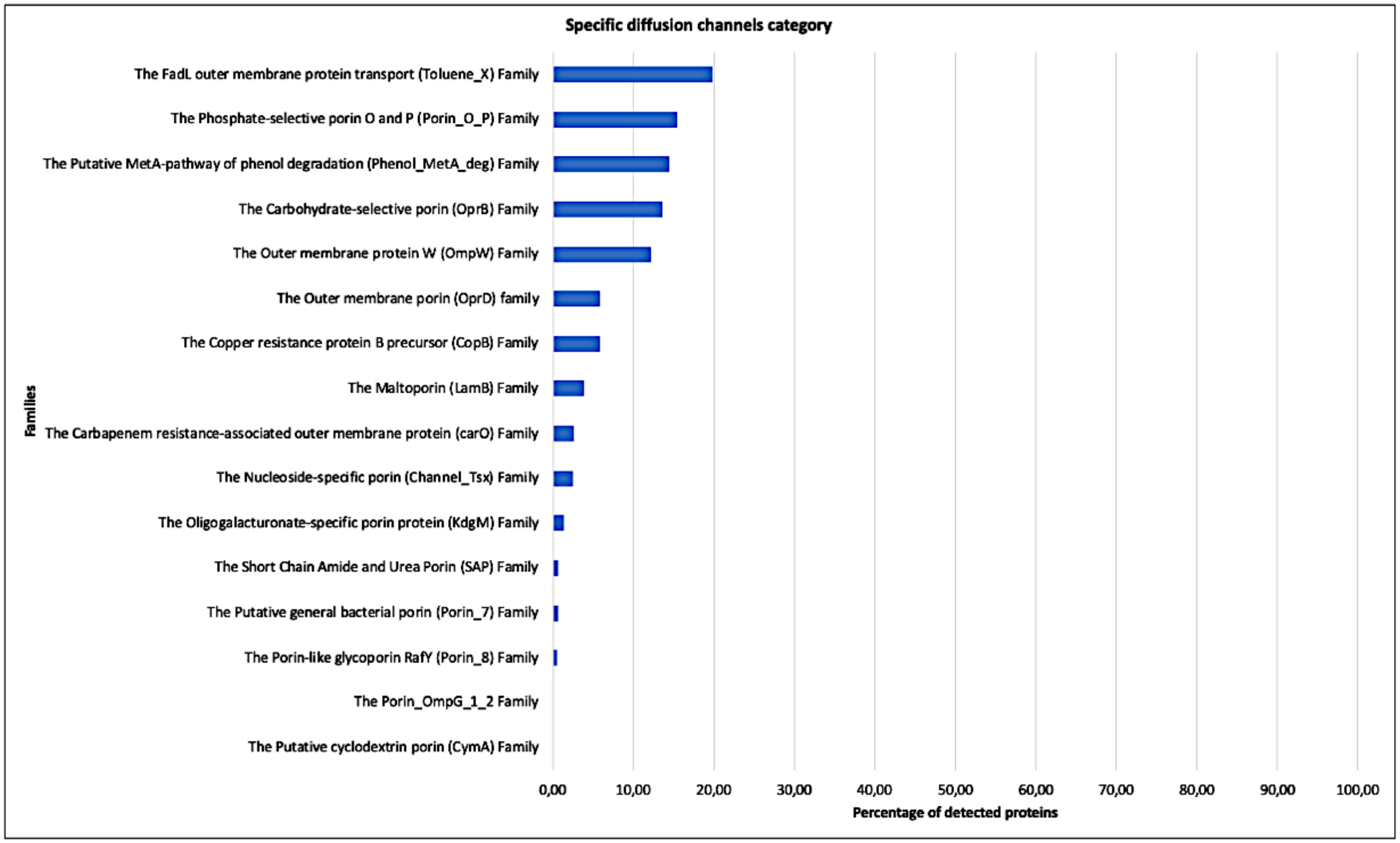

Supplementary Figure 4. The fraction of detected proteins in each of the families belonging to 'specific diffusion channels' category.

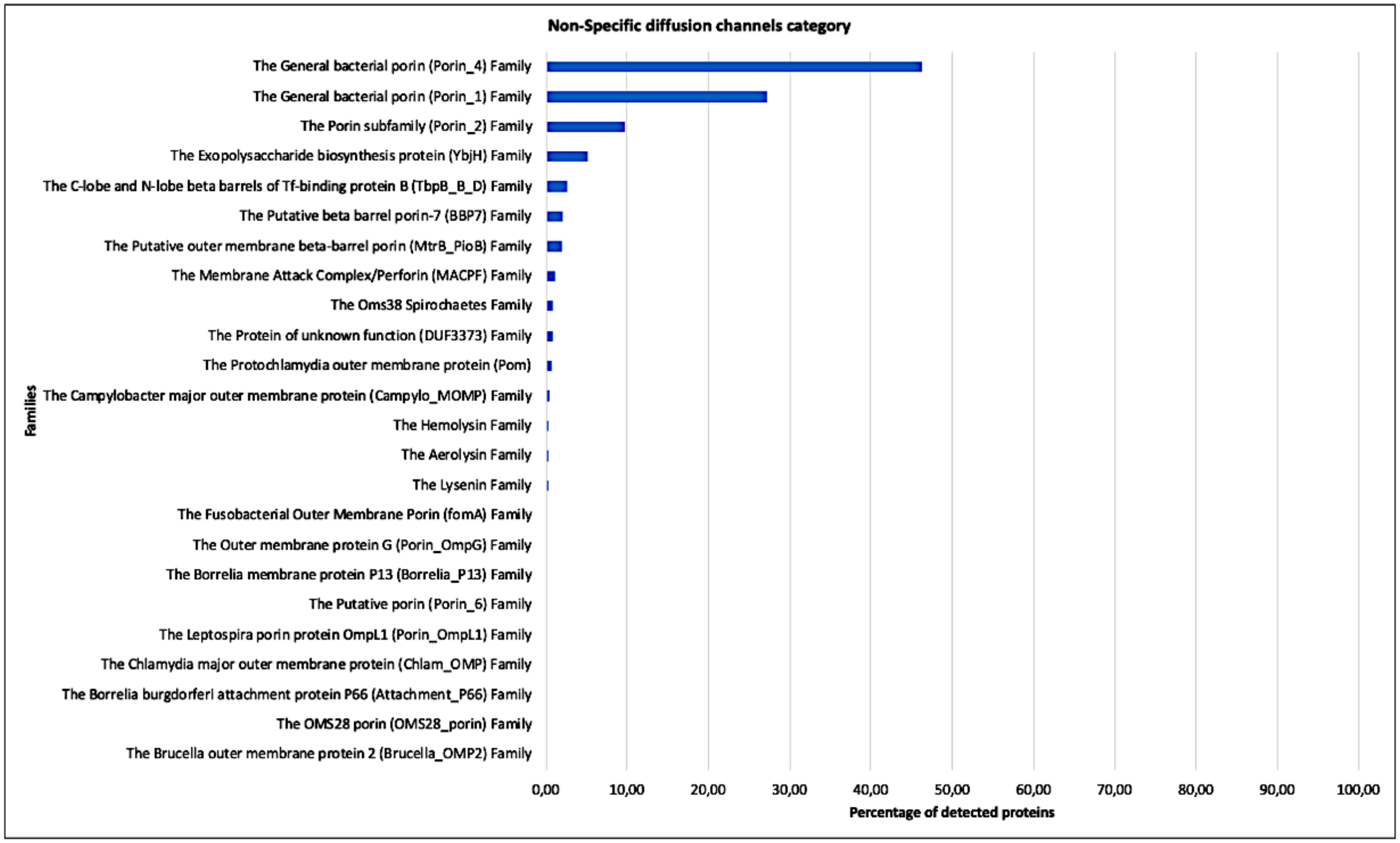

Supplementary Figure 5. The fraction of detected proteins in each of the families belonging to 'non-specific diffusion channels' category.

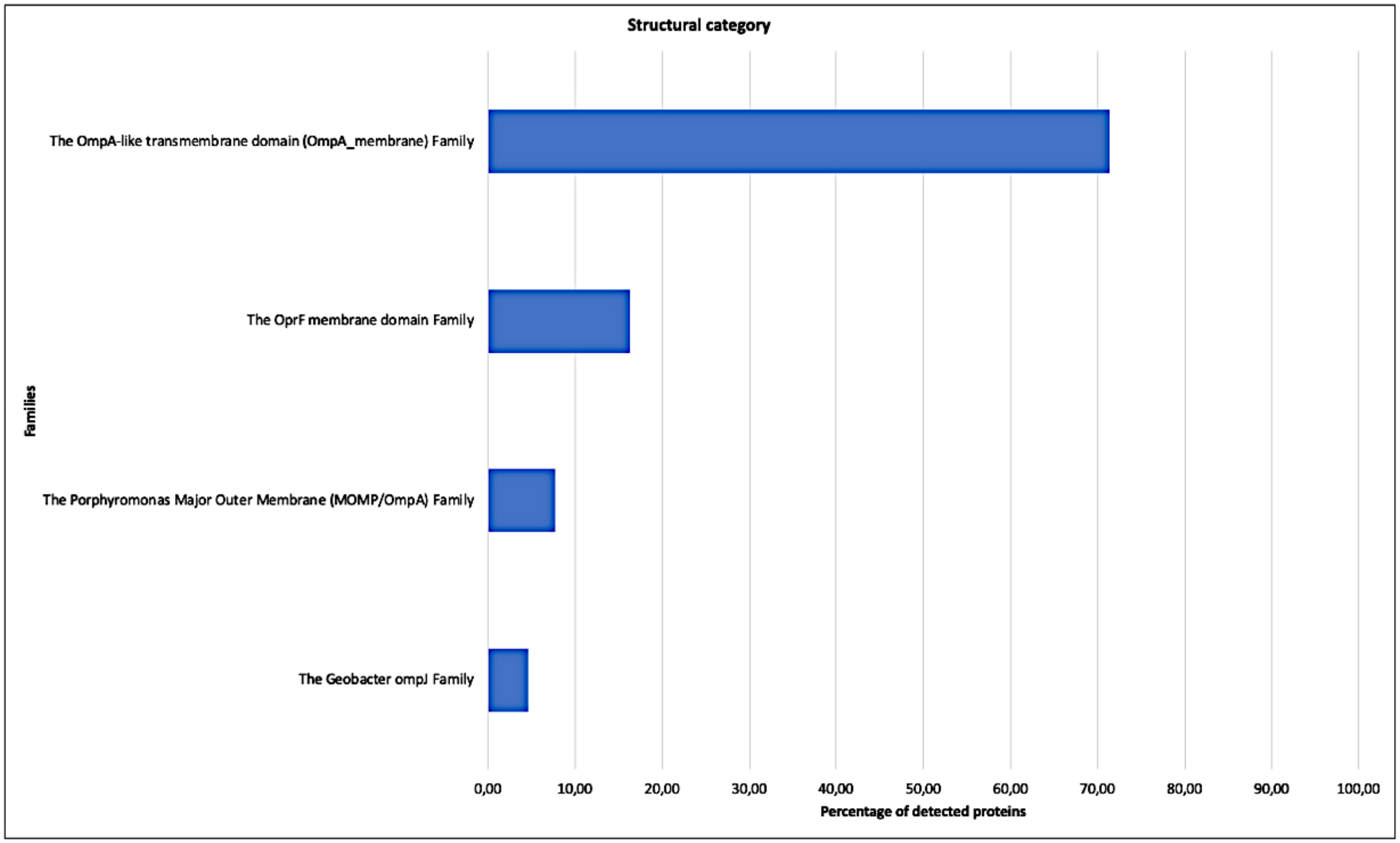

Supplementary Figure 6. The fraction of detected proteins in each of the families belonging to 'structural' category.

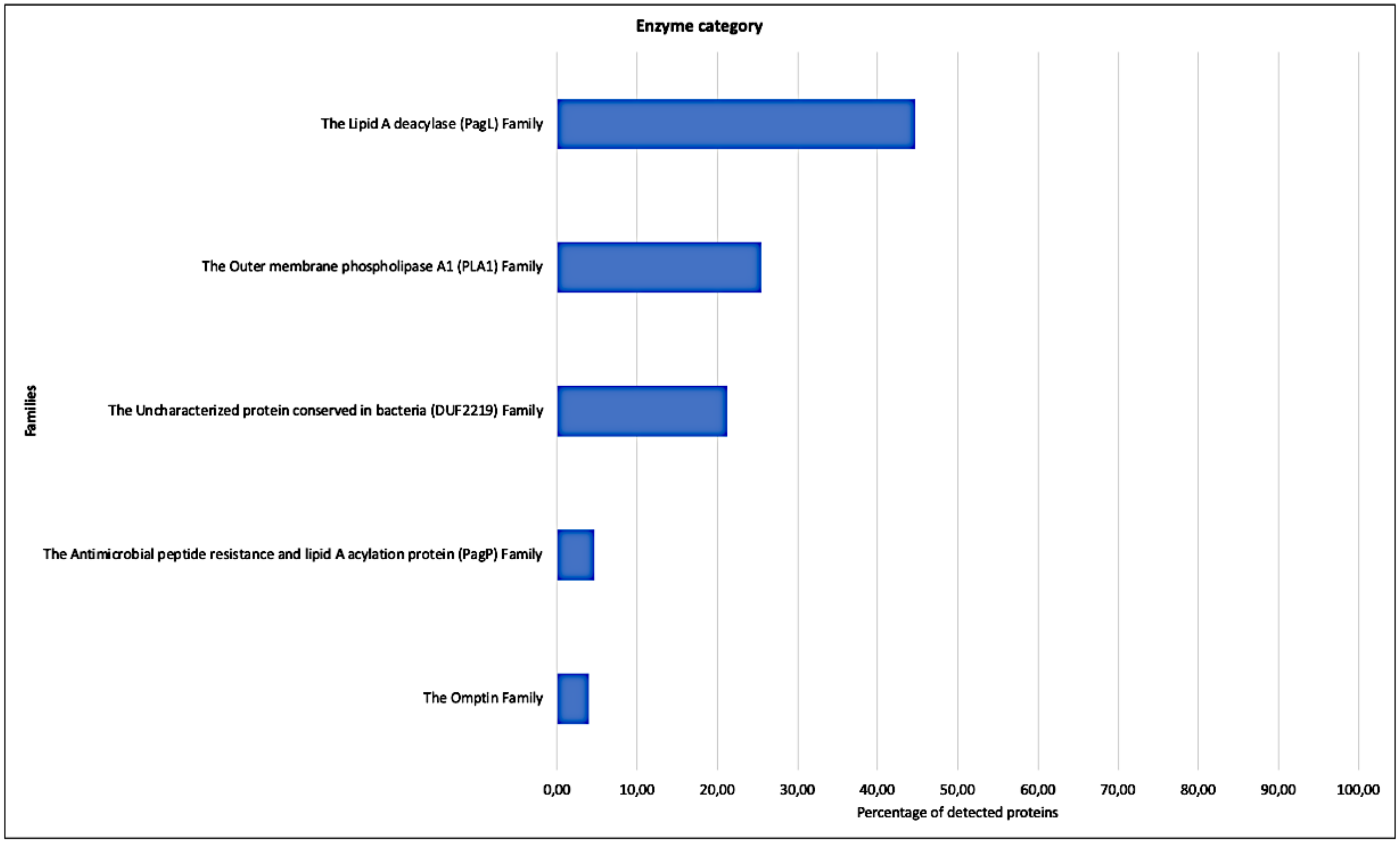

Supplementary Figure 7. The fraction of detected proteins in each of the families belonging to 'enzymes' category.

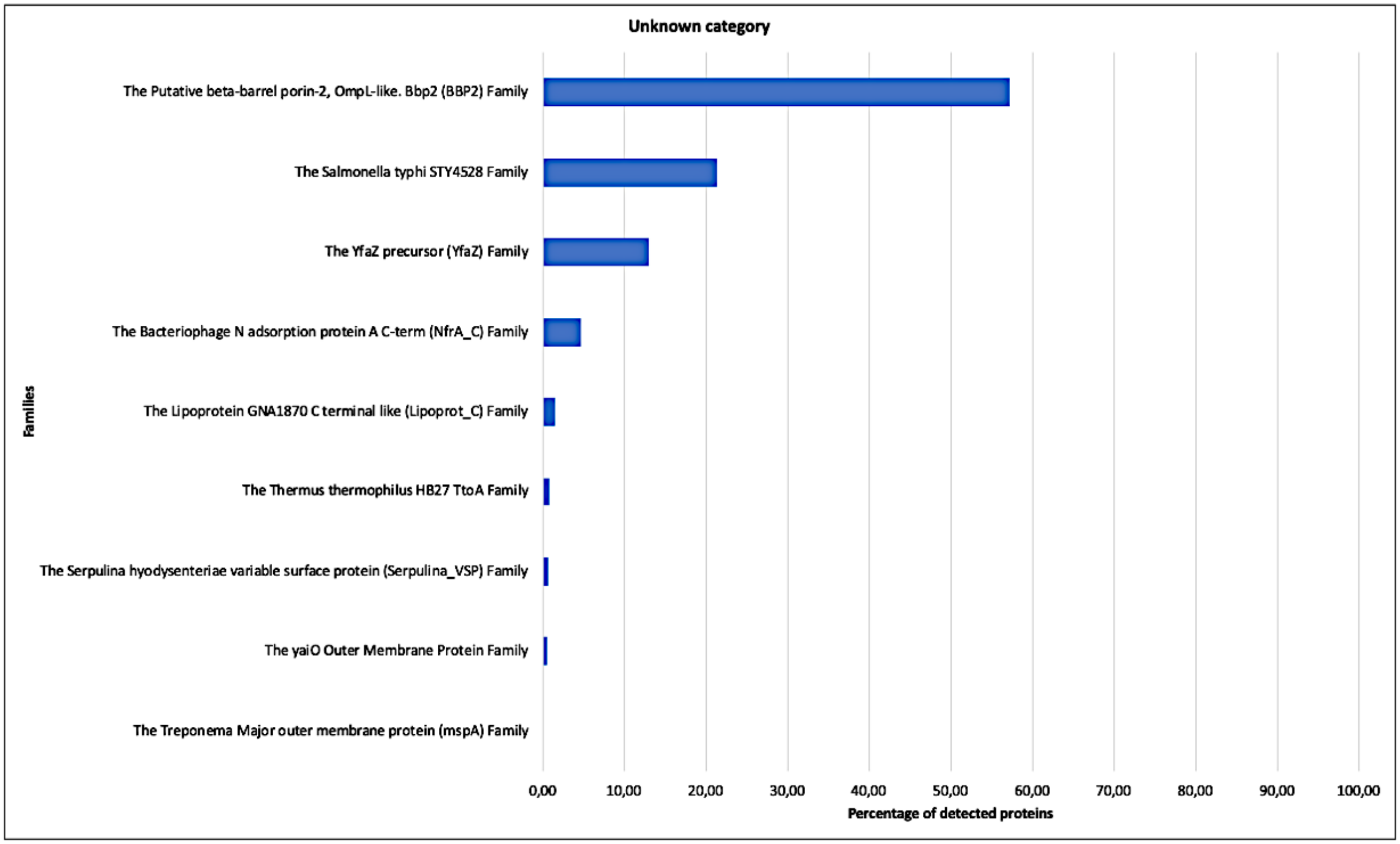

Supplementary Figure 8. The fraction of detected proteins in each of the families belonging to 'unknown' category.

1.2 Supplementary Tables

Supplementary Table 1. List of domain of unknown function families (DUF families)

The Family name	PFAM domain	Function	Inclusion in the genome search	Inclusion in the OMPdb
The UPF0164 Family	PF03687	Unknown	No	Yes
The Porin_5 Family	PF16930	Unknown	No	Yes
The BBP2_2 Family	PF10082	Unknown	No	Yes
The Outer membrane protein beta-barrel Family	PF14905	Unknown	No	Yes
The F plasmid transfer operon (TraF) Family	PF13729	Unknown	No	Yes
The Porin10 Family	PF14121	Unknown	No	Yes
The Gcw_chp Family	PF09694	Unknown	No	Yes
The Putative OmpA-OmpF-like porin Family	PF16961	Unknown	No	Yes
The DUF481 Family	PF04338	Unknown	No	Yes
The DUF3308 Family	PF10677	Unknown	No	Yes
The DUF2490 Family	PF10895	Unknown	No	Yes
The DUF2715 Family	PF11276	Unknown	No	Yes
The DUF3078 Family	PF11336	Unknown	No	Yes
The DUF3138 Family	PF11383	Unknown	No	Yes

The DUF3187 Family	PF12099	Unknown	No	Yes
The DUF3575 Family	PF14391	Unknown	No	Yes
The DUF4421 Family	PF15283	Unknown	No	Yes
The DUF4595 Family	PF16412	Unknown	No	Yes
The DUF5777 Family	PF19089	Unknown	No	Yes
